# FMA-MADDPG: Constrained Multi-Agent Resource Optimization with Channel Prediction in 6G Non-Terrestrial Networks

**DOI:** 10.3390/s26010148

**Published:** 2025-12-25

**Authors:** Chunyu Yang, Kejian Song, Jing Bai, Cuixing Li, Yang Zhao, Zhu Xiao, Yanhong Sun

**Affiliations:** 1 School of Astronautics, Harbin Institute of Technology, Harbin 150001, China; 22b918170@stu.hit.edu.cn (C.Y.); yangzhao@hit.edu.cn (Y.Z.); 2 The 54th Research Institute, China Electronics Technology Group Corporation, Shijiazhuang 050081, China; sunyu861226@163.com; 3 School of Artificial Intelligence, Key Laboratory of Intelligent Perception and Image Understanding, Ministry of Education, Xidian University, Xi’an 710071, China; 24171213931@stu.xidian.edu.cn (K.S.); 24171214002@stu.xidian.edu.cn (C.L.); 4 College of Computer Science and Electronic Engineering, Hunan University, Changsha 410082, China; zhxiao@hnu.edu.cn

**Keywords:** resource allocation, deep reinforcement learning, non-terrestrial networks, low-Earth-orbit satellites, channel state prediction, remote sensing applications

## Abstract

Sixth-generation (6G) wireless systems aim to integrate terrestrial, aerial, and satellite networks to support large-scale remote sensing and service delivery. In such non-terrestrial networks (NTNs), channels change quickly and the multi-tier architecture is heterogeneous, which makes real-time channel state acquisition and cooperative resource scheduling difficult. This paper proposes an FMA-MADDPG framework that combines a channel prediction module with a constraint-based multi-agent deep deterministic policy gradient scheme. The Fusion of Mamba and Attention (FMA) predictor uses a Mamba state-space backbone and a multi-head self-attention block to learn both long-term channel evolution and short-term fluctuations, and forecasts future CSI. The predicted channel information is added to the agents’ observations so that scheduling decisions can take expected channel variations into account. A constraint-based reward is also designed, with explicit performance thresholds and anti-idle penalties, to encourage fairness, avoid free-riding, and promote cooperation among heterogeneous agents. In a representative NTN uplink scenario, the proposed method achieves higher total reward, efficiency, load balance, and cooperation than several DRL baselines, with relative gains around 10–20% on key metrics. These results indicate that prediction-aware cooperative reinforcement learning is a useful approach for resource optimization in future 6G NTN systems.

## 1. Introduction

Sixth-generation (6G) wireless communication is reshaping how networks are designed and operated [1]. Unlike fifth-generation (5G) wireless communication—which mainly targets terrestrial users and base stations—6G aims to build a global network that connects ground, air, and space. This development is closely linked to Earth observation and remote sensing. In these applications, large volumes of heterogeneous data are generated by distributed ground sensors, unmanned aerial vehicles (UAVs), and satellites. Typical missions such as environmental monitoring, disaster response, precision agriculture, and climate analysis require high data rates, low latency, and broad spatial coverage [2].

Current terrestrial networks are not well suited for large-scale remote sensing tasks [3]. In remote regions such as oceans, deserts, forests, and mountains, deploying dense ground infrastructure is often too costly or technically infeasible. Even in urban areas, multipath fading, building blockage, and congestion frequently degrade communication quality. These limitations create a mismatch between front-end data acquisition and back-end data processing. As a result, the timeliness and reliability of geospatial services are reduced.

Non-terrestrial networks (NTNs) have been introduced to bridge this gap. NTNs integrate low-Earth-orbit (LEO) satellites, high-altitude platforms (HAPs), and terrestrial networks into a three-dimensional architecture [4]. In this structure, user equipment (UE) represents ground sensors, UAVs, or mobile terminals that generate data. HAPs operate as relay and edge computing nodes that support local processing, data fusion, and temporary storage. LEO satellites provide wide-area backhaul and global coordination so that data from distributed sensors can be efficiently aggregated and delivered [5]. Together, these three layers form a cooperative communication and computing system capable of supporting data-intensive sensing tasks across large geographic regions.

Deploying and operating such an NTN architecture introduces several technical challenges. First, wireless channels between UEs, HAPs, and LEO satellites vary rapidly. Atmospheric turbulence, rain, blockages, and Doppler shifts cause fast and sometimes correlated changes in signal quality. The UE–HAP and HAP–LEO links also differ in propagation characteristics, which increases uncertainty. Under these conditions, obtaining accurate channel state information (CSI) in real time becomes difficult. Without reliable CSI, network agents must make scheduling decisions based on partial observations, often resulting in poor resource utilization. Second, LEO satellites move quickly along their orbits, causing continuous changes in network topology and connectivity [6]. The available bandwidth, computing resources, and relay paths in a given area depend on which satellites are visible at that moment. This makes multi-agent resource coordination a time-varying problem. Traditional optimization methods often assume fixed topologies, convex formulations, or full system knowledge, and therefore struggle under such dynamics. Third, reinforcement learning (RL) and multi-agent RL (MARL) methods face their own limitations. Many RL algorithms assume stationary environments or full observability, which is unrealistic in NTNs. Even with partial observability, learning can be unstable when state representations are weak or rewards are ambiguous. Without domain-specific prediction or augmentation, policies may become short-sighted and fail to generalize across agents, locations, and time scales [7].

In this work, we propose a unified framework, termed Fusion-of-Mamba-and-Attention-enhanced Multi-Agent Deep Deterministic Policy Gradient (FMA-MADDPG), that combines predictive modeling, constraint-aware learning, and coordinated control across the three-layer UE–HAP–LEO NTN. To improve channel observability, we design a prediction module named FMA (Fusion of Mamba and Attention). It employs Mamba blocks to capture long-term channel evolution and multi-head self-attention to model short-term variations. Through this design, FMA can forecast CSI several steps ahead. The predicted CSI is incorporated into the observations of HAP and LEO agents, enabling their decisions to reflect both current and near-future channel conditions. In parallel, we develop a constrained multi-agent reinforcement learning scheme based on multi-agent deep deterministic policy gradient (MADDPG) within a centralized training and decentralized execution (CTDE) framework. The goal is not only to increase throughput but also to meet constraints related to energy usage, task completion, and cooperation. A structured reward is introduced to connect local actions to global stability and to reduce issues such as reward exploitation and policy collapse.

We evaluate the proposed framework in a simulation environment that reflects key operational characteristics of NTNs. The environment includes dynamic user traffic, fading channels, HAP mobility, LEO orbital motion, and interactions among multiple agents. Several channel prediction models and MARL baselines are incorporated for comparison. Experimental results show that the proposed FMA-MADDPG method improves throughput, reduces delay, and enhances coordination efficiency relative to these baselines.

In summary, this work contributes to NTN research by integrating channel prediction, constraint-aware multi-agent learning, and multi-layer cooperation within a single framework. Existing studies typically focus on either channel prediction or RL-based resource allocation in isolation, or combine standard predictors (such as RNNs, CNNs, or Transformers) with RL in terrestrial or simplified satellite scenarios. In contrast, our framework advances NTN design in three main aspects. First, at the architectural level, we introduce the FMA model, which integrates a Mamba state-space backbone, multi-head attention, and attention-based temporal pooling to capture multi-scale channel dynamics and to improve decision robustness. Second, at the learning-strategy level, we embed this long-context predictor into a constraint-aware MARL scheme based on MADDPG with CTDE. Predicted CSI augments agent observations, while fairness and delay are incorporated through a shaped reward. Third, at the system-design level, we construct and evaluate a joint prediction-and-scheduling solution for a realistic three-layer UE–HAP–LEO NTN, rather than optimizing prediction and resource allocation separately. By addressing channel uncertainty, partial observability, and constrained optimization within this combined prediction–MARL setting, the proposed system provides a practical approach for improving resource allocation in future 6G NTNs.

## 2. Related Works

The rapid development of NTNs has attracted growing interest in intelligent and adaptive communication under dynamic and resource-limited conditions. In such systems, two related problems are central. The first is how to design distributed but coordinated decision policies for resource scheduling [8]. The second is how to sense or predict time-varying channels, since CSI quality directly affects what each agent can observe and how well it can learn [9]. Most existing studies address only one of these problems. They either apply deep reinforcement learning (DRL) to optimize resource allocation, or use sequential models for channel estimation, but they do not treat prediction and control together in the same NTN framework. In this section, we briefly review DRL-based scheduling and channel prediction work that is relevant to our setting.

### 2.1. Deep Reinforcement Learning for Resource Scheduling

Single-agent DRL methods such as DQN, DDPG [10], and PPO have been applied to power control, spectrum sharing, and task offloading. However, these methods face notable limitations in systems with many users or distributed controllers. As the number of agents grows, the environment becomes highly non-stationary and the state information becomes only partially observable, making single-agent DRL difficult to scale.

Multi-agent reinforcement learning (MARL) addresses these issues by letting multiple agents learn policies jointly. Algorithms such as MADDPG, QMIX, and MAPPO support decentralized execution while using centralized critics or shared information during training [11]. In wireless communication, MARL has been applied to mobile edge computing (MEC), UAV-assisted networks, and vehicular networks. In NTNs, however, MARL still faces several difficulties. The network topology changes due to satellite motion, channel quality is strongly affected by fading and blockage, and reward signals may be delayed or sparse [12]. In addition, many MARL schemes focus on a single metric, for example throughput or delay. They often do not model constraints that are important in NTNs, such as fairness among users, energy usage, or explicit cooperation between different layers. This limits their applicability in hierarchical UE–HAP–LEO systems.

### 2.2. Channel Prediction in Dynamic Wireless Environments

Accurate and timely CSI is important for good scheduling decisions, especially in mobile systems with fast channel variations. In NTNs, Doppler shifts and moving platforms make this problem even more pronounced. Traditional model-based estimators, such as autoregressive (AR) models, Kalman filters, and least-squares interpolation [13], can work well in slowly changing channels. They often perform poorly, however, when fading is fast, nonlinear, or strongly frequency-selective.

Data-driven methods based on deep learning have improved channel prediction in many wireless settings. Recurrent neural networks (RNNs), including long short-term memory (LSTM) and gated recurrent unit (GRU) models, are widely used to capture temporal correlations [14]. Later, hybrid architectures and temporal convolutional networks (TCNs) were introduced to extend the receptive field and reduce training cost. Attention-based models, such as the Transformer, further improved long-range sequence modeling, but they often require high computational resources when the sequence is long.

More recently, state-space sequence models (SSMs) such as S4 and its efficient variant Mamba [15] have been proposed. Mamba can model very long sequences with structured state spaces and linear complexity in the sequence length. This makes it a promising tool for NTN channel prediction, where long time windows are needed to capture slow orbital dynamics together with fast fading effects. However, the use of such long-context state-space models for NTN channel prediction is still limited in the current literature.

A common limitation in most channel prediction work is that prediction is treated as an isolated task, separate from resource allocation or control [16]. In NTNs, where agents’ capacity to perceive the environment is constrained by channel conditions, this separation is problematic. It is more natural to feed predicted CSI directly into the learning loop, so that agents can use both current and anticipated future states when choosing actions. Only a few studies attempt such a tight integration [17]. In addition, NTN channels show multi-scale behavior (fast fading versus slower orbital motion) and irregular degradation (such as sudden blockage). This calls for predictors that can extract fine-grained features and at the same time remain stable over long time horizons. Existing single-structure models do not fully address this need and motivate hybrid designs. To the best of our knowledge, no prior work for NTNs combines a state-space-based channel predictor, prediction-augmented multi-agent RL, and explicit constraint-shaped rewards in a unified three-layer UE–HAP–LEO architecture.

## 3. Methodology

This section introduces the proposed NTN uplink optimization framework. We first describe the system architecture and channel models. We then give the problem formulation. After that, we explain how the Fusion of Mamba and Attention (FMA) channel prediction model is integrated with the MADDPG-based multi-agent scheduling scheme.

### 3.1. System Architecture and Network Model

**Figure 1:**
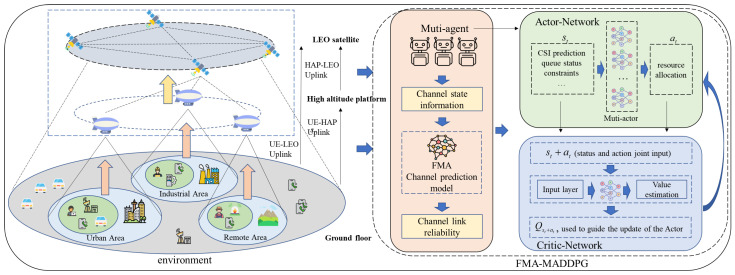
Illustration of the proposed NTN uplink architecture and the integration of the FMA-enhanced multi-agent decision framework. The left side shows the three-layer UE–HAP–LEO communication scenario across urban, industrial, and remote areas, while the right side highlights the actor–critic network design, where predicted CSI from the FMA module is embedded into multi-agent decision making for resource allocation.

As shown in Figure 1, we consider an NTN uplink with two types of links: UE–HAP (uplink 1) and HAP–LEO (uplink 2). UEs send data to HAPs using OFDM. HAPs then forward the aggregated traffic to LEO satellites using NOMA. This layered design reflects the different roles of the ground, air, and space segments, and allows resource management to be coordinated across tiers. The right-hand side of Figure 1 shows how the FMA-based channel predictor and the multi-agent actor–critic network are combined, with predicted CSI fed into the decision module for resource allocation.

The system includes a set H={1,…,H} of HAPs deployed at identical altitudes. Each HAP covers a sub-area and serves a subset of UEs denoted by Z={1,…,Z}. This assumption simplifies the geometry and reflects typical HAP deployment where platforms operate in a narrow altitude band. The sub-areas are represented by A={1,…,A}. The 3D position of HAP *h* is $d^{\mathrm{HAP}}=\left\{x_{h},y_{h},t_{h}\right\}$. Within each sub-area, UEs are randomly distributed and transmit data to their associated HAPs using the available sub-channel resources [18].

Each HAP is allocated an equal share of orthogonal sub-channels under OFDM. The total bandwidth is divided into $|C_{Z}|$ orthogonal sub-channels. At each time slot k∈K, where K={1,…,K}, the bandwidth assigned to the link between UE *z* and HAP *h* is

(1) $$B_{z,h}\left(k\right)=\frac{B_{\mathrm{Tot}}^{\mathrm{UE}–\mathrm{HAP}}}{|C_{Z}|},$$

subject to

$$\sum_{z=1}^{Z}B_{z,h}\left(k\right)\leq B_{\mathrm{Tot}}^{\mathrm{UE}–\mathrm{HAP}},$$

where $B_{\mathrm{Tot}}^{\mathrm{UE}–\mathrm{HAP}}$ is the total bandwidth for all UE–HAP links and $C_{Z}=\left\{1,\ldots ,C_{\mathrm{Tot}}\right\}$ is the set of sub-channels. In most cases the number of UEs exceeds the number of sub-channels, i.e., $\left|Z|>\right|C_{Z}|$, so an efficient allocation strategy is needed to avoid congestion.

The space segment is modeled by a set L={1,…,L} of LEO satellites that form a constellation. Each satellite has a sub-channel index set $Y=\{1,\ldots ,Y_{\mathrm{tot}}\}$, where $Y_{\mathrm{tot}}$ is the number of sub-channels in uplink 2. The resources assigned to each HAP are denoted by $J^{R}=\left\{J_{1},\ldots ,J_{h},\ldots ,J_{H}\right\}$. Each LEO satellite also supports a set $\vartheta^{l}=\left\{1,\ldots ,r_{\mathrm{tot}}^{l}\right\}$ of sub-carriers, where $r_{\mathrm{tot}}^{l}$ is the total number of sub-carriers for satellite *l*. UEs first send data to HAPs, and HAPs then forward the data to the LEO satellites using NOMA. The satellites apply successive interference cancellation (SIC) to decode the superimposed signals from different HAPs. For simplicity, each UE is assumed to have a single antenna.

The UE–HAP link uses OFDM. OFDM is robust to multipath fading and is widely used in terrestrial links, which makes it suitable for dense terrestrial–aerial communication [18]. For the HAP–LEO link, we use NOMA. NOMA can handle heterogeneous traffic and varying user densities and can increase spectral efficiency by power-domain multiplexing. LEO satellites are assumed to have sufficient processing capability to run SIC and separate the received signals from multiple HAPs. Although NOMA increases receiver complexity, it is a reasonable choice for HAP–LEO links, since the processing can be done on the satellite side [19]. By adjusting transmit power levels of different HAPs, NOMA can also improve fairness, because nodes with better channels can use less power, while others can be assigned more power when needed, without reducing overall system efficiency.

In our model, multiple HAPs sharing the same HAP–LEO sub-channel are multiplexed in the power domain. At the LEO receiver, their superimposed signals are ordered according to the effective channel gains and decoded successively using SIC, assuming that the resulting signal-to-interference-plus-noise ratios are sufficient for reliable detection. After decoding, the contribution of each recovered HAP signal is subtracted from the composite received signal before decoding the next one, and any residual interference is treated as noise in the achievable-rate calculations. The channel prediction module focuses on forecasting the large-scale and small-scale gains of the individual HAP–LEO links; the aggregate interference level on a shared sub-channel is implicitly determined by the simultaneous transmissions from the scheduled HAPs.

### 3.2. Channel Models

The position of the *z*-th UE associated with the *h*-th HAP at time slot *k* is

$$d^{\mathrm{UE}}\left(k\right)=\left\{x_{z}\left(k\right),y_{z}\left(k\right),t_{z}\left(k\right)\right\}.$$

Each UE has an initial traffic demand $D_{z}$ to be transmitted. We use $\varphi_{h,z}\left(k\right)\in \left\{0,1\right\}$ to denote the association indicator between UE *z* and HAP *h* at time *k*:

(2) $$\varphi_{h,z}\left(k\right)=\left\{\begin{array}{cc} 1, & \mathrm{if}\;\mathrm{UE}\;z\;\mathrm{is}\;\mathrm{associated}\;\mathrm{with}\;\mathrm{HAP}\;h,\\ 0, & \mathrm{otherwise}. \end{array}\right.$$

For simplicity, we assume that UEs are static during an episode. This matches many remote sensing scenarios with fixed ground sensors and allows us to focus on NTN-specific channel dynamics. Each UE can be associated with at most one HAP in a time slot, so we have

(3) $$\sum_{h\in H}\varphi_{h,z}\left(k\right)\leq 1,\;\forall z\in Z,\forall k\in K.$$

Power allocation is another key factor in the UE–HAP link. Let $P={\left[P_{1},\ldots ,P_{Z}\right]}^{T}$ be the vector of transmit powers for all UEs. We write the allocation compactly as

(4) $$P={\left[P_{1},\ldots ,P_{Z}\right]}^{T},\;\forall z\in Z,$$

and constrain the link-level power by

$$0\leq P_{\left(z,h\right)}\left(k\right)\leq P_{z}^{\max}.$$

This means that the transmit power from UE *z* to HAP *h* at time *k* cannot exceed the hardware limit $P_{z}^{\max}$ and cannot be negative.

The instantaneous SNR at HAP *h* from UE *z* at time slot *k* is modeled as

(5) $$\theta_{\left(z,h\right)}\left(k\right)=\frac{\varphi_{h,z}\left(k\right)R_{cf}P_{\left(z,h\right)}\left(k\right)G_{\left(z,h\right)}\left(k\right)T_{h}\left(k\right)F_{h}}{C_{B}B_{z,h}\left(k\right)Ts_{n}},$$

where $G_{\left(z,h\right)}\left(k\right)$ and $T_{h}\left(k\right)$ are the transmit and receive antenna gains, $F_{h}$ represents the path loss term (including free-space loss and large-scale attenuation), and $R_{cf}$ captures small-scale fading on the UE–HAP link. The factor $C_{B}Ts_{n}$ combines the noise spectral density and receiver temperature, and $B_{z,h}\left(k\right)$ is the bandwidth assigned to this link. Equation (5) thus follows a standard link-budget form for the air-to-ground channel. In this expression, $\varphi_{h,z}\left(k\right)\in \left\{0,1\right\}$ is an association indicator that guarantees that the SNR is zero whenever UE *z* is not served by HAP *h* at slot *k*. The coefficient $R_{cf}$ models small-scale fading on the UE–HAP link and is drawn from a Rayleigh block-fading process with unit average power. The product $G_{\left(z,h\right)}\left(k\right)T_{h}\left(k\right)F_{h}$ aggregates the transmit and receive antenna gains and the large-scale path loss, which is computed from a standard air-to-ground link budget including free-space loss and log-normal shadowing. The denominator $C_{B}Ts_{n}B_{z,h}\left(k\right)$ corresponds to the thermal noise power over the allocated bandwidth, given a receiver temperature *T* and noise spectral density $s_{n}$.

Given the SNR, the instantaneous rate on the UE–HAP link is

(6) $$\Phi_{\left(z,h\right)}\left(k\right)=B_{z,h}\left(k\right)\log_{2}\left[1+\theta_{\left(z,h\right)}\left(k\right)\right],$$

where the Shannon formula is used as an upper bound on achievable rate. Under the equal-bandwidth allocation in (1), this expression reduces to

(7) $$\Phi_{\left(z,h\right)}\left(k\right)=\frac{B_{\mathrm{Tot}}^{\mathrm{UE}–\mathrm{HAP}}}{|C_{Z}|}\log_{2}\left[1+\theta_{\left(z,h\right)}\left(k\right)\right].$$

Here $B_{\mathrm{Tot}}^{\mathrm{UE}–\mathrm{HAP}}$ is the total bandwidth for all UE–HAP links and $|C_{Z}|$ is the number of sub-channels.

To evaluate fairness among users, we adopt Jain’s fairness index:

(8) $$I_{f}\left(k\right)=\frac{{\left[\sum_{z=1}^{Z}{\hat{\Gamma }}_{z}\left(k\right)\right]}^{2}}{\left|Z\right|\sum_{z=1}^{Z}{\left[{\hat{\Gamma }}_{z}\left(k\right)\right]}^{2}},$$

where ${\hat{\Gamma }}_{z}\left(k\right)$ denotes the cumulative throughput of UE *z* up to time slot *k*. If all UEs obtain similar cumulative throughput, the index $I_{f}\left(k\right)$ is close to 1; if a few UEs dominate, the index drops towards 0. This index is used later in the reward function to encourage balanced service across UEs.

We also include end-to-end latency in the channel model. The delay for data from UE *z* to HAP *h* at time *k* is

(9) $$\varpi_{\left(z,h\right)}\left(k\right)=\frac{D_{z}}{\Phi_{\left(z,h\right)}\left(k\right)}+\Delta \left(\mu -1\right)+\frac{\zeta_{\left(z,h\right)}\left(k\right)}{C_{l}},$$

where $D_{z}/\Phi_{\left(z,h\right)}\left(k\right)$ is the transmission time for payload $D_{z}$, Δ(μ−1) models queuing delay, and $\zeta_{\left(z,h\right)}\left(k\right)/C_{l}$ is the propagation delay on the UE–HAP path. This expression exposes the trade-off between rate, queuing delay, and propagation delay. It also shows that throughput, fairness, and latency must be considered together when designing the NTN resource allocation policy [20]. The above channel and delay models deliberately abstract away several propagation effects that may be present in real NTN deployments. Examples include detailed atmospheric absorption and scintillation, rain attenuation at higher frequency bands, Earth curvature over very long links, and spatial correlation among neighboring links and beams. We adopt this intermediate level of fidelity in order to preserve the dominant large-scale geometry, mobility-induced path loss variation, and small-scale fading dynamics that are critical for evaluating learning-based schedulers, while keeping the state dimension and parameter space manageable for extensive multi-agent training and ablation studies. Consequently, the absolute performance values reported in Section 4 should not be interpreted as predictions for a particular commercial system, but rather as relative comparisons among different prediction and control schemes under the same controlled conditions. Extending the framework to standardized NTN channel models and richer propagation phenomena is left for future work.

Based on the above network and channel models, we formulate the uplink optimization problem as a joint resource allocation task. At each time slot *k*, we define a scalar system reward $R_{\mathrm{tot}}\left(k\right)$ that aggregates several performance aspects, including total capacity, average end-to-end delay, Jain’s fairness index, and the number of cooperation events between HAPs and LEOs. The detailed form of this composite reward and the corresponding normalization are given in Section 3.3, see (13). Formally, the optimization problem is to maximize the cumulative system reward over a finite horizon under UE–HAP association constraints, power limits, sub-channel availability, fairness requirements, and latency thresholds. The constraints are given by the association rules in (2)–(3), the power constraints in (4), the capacity relations in (6)–(7), the fairness index in (8), and the latency bound in (9). Because the problem mixes binary association variables with continuous power variables and includes nonlinear rate and delay terms, it falls into the class of mixed-integer nonlinear programming (MINLP), which is NP-hard and difficult to solve for large NTN systems. Over a finite horizon of *K* time slots, the corresponding optimization problem can be written compactly as

$$\begin{array}{cc} \max_{\left\{\varphi_{h,z}\left(k\right)\right\},\;\left\{P_{\left(z,h\right)}\left(k\right)\right\}}\;\; & \sum_{k=1}^{K}R_{\mathrm{tot}}\left(k\right)\\ s.t.\;\; & \varphi_{h,z}\left(k\right)\in \left\{0,1\right\},\;\forall h\in H,\;z\in Z,\;k\in K,\\ \; & \sum_{h\in H}\varphi_{h,z}\left(k\right)\leq 1,\;\forall z\in Z,\;k\in K,\\ \; & 0\leq P_{\left(z,h\right)}\left(k\right)\leq P_{z}^{\max},\;\forall h\in H,\;z\in Z,\;k\in K,\\ \; & \sum_{z\in Z}B_{z,h}\left(k\right)\leq B_{\mathrm{Tot}}^{\mathrm{UE}–\mathrm{HAP}},\;\forall h\in H,\;k\in K,\\ \; & \Phi_{\left(z,h\right)}\left(k\right)\;\mathrm{given}\;\mathrm{by}\;\left(6)–(7\right),\;\forall h\in H,\;z\in Z,\;k\in K,\\ \; & I_{f}\left(k\right)\geq I_{f}^{\min},\;\forall k\in K,\\ \; & \varpi_{\left(z,h\right)}\left(k\right)\leq \varpi^{\max},\;\forall h\in H,\;z\in Z,\;k\in K, \end{array}$$

where $R_{\mathrm{tot}}\left(k\right)$ denotes the composite system reward at slot *k*, $I_{f}^{\min}$ is a target fairness level, and $\varpi^{\max}$ is the maximum tolerable end-to-end delay. The association constraints ensure that each UE is connected to at most one HAP per slot, the power and bandwidth constraints enforce hardware and spectral limits, and the fairness and delay constraints reflect service quality requirements. Together with the nonlinear rate and delay relations in 6–9, this yields a mixed-integer nonlinear program that is difficult to solve optimally in real time for large NTN systems.

To handle these difficulties, we do not solve this mixed-integer nonlinear program directly at run time. Instead, we adopt a predictive and learning-based approach. Channel state information is first estimated and predicted by the FMA module, and the predicted channel evolution is then used to guide decisions in a multi-agent deep reinforcement learning framework [21]. In this framework, the original optimization problem is cast as a Markov decision process: the state contains the (predicted) channel features and queue information, the actions represent association and power control decisions, and the reward reflects the composite objective $R_{\mathrm{tot}}\left(k\right)$.

### 3.3. DRL and Training Model for Multi-Agents

In the proposed three-layer space–air–ground network, the resource allocation problem involves many UEs, HAPs, and LEO satellites. The links have different characteristics, the traffic is time-varying, and the wireless channels change over time. Each network node needs to make sequential decisions on UE association, bandwidth allocation, and power control in a distributed but still coordinated way [22]. This naturally leads to a multi-agent decision-making problem. We model it as a partially observable Markov game (POMG) defined by

(10) $$G=\langle N,𝒮,{\left\{{𝒪}_{i}\right\}}_{i\in N},{\left\{A_{i}\right\}}_{i\in N},P,{\left\{r_{i}\right\}}_{i\in N},\gamma \rangle ,$$

where N={1,2,…,N} is the set of agents, 𝒮 is the global state space, ${𝒪}_{i}$ is the local observation of agent *i*, $A_{i}$ is its action space, *P* is the state transition function, $r_{i}$ is the reward for agent *i*, and γ∈(0,1) is the discount factor.

At time slot *t*, the global state is

(11) $$s_{t}=\left[H_{t}^{\mathrm{UE}-\mathrm{HAP}},H_{t}^{\mathrm{HAP}-\mathrm{LEO}},B_{t},P_{t},Q_{t}\right],$$

where $H_{t}^{\mathrm{UE}-\mathrm{HAP}}$ and $H_{t}^{\mathrm{HAP}-\mathrm{LEO}}$ are the CSI matrices of the UE–HAP and HAP–LEO links, $B_{t}$ is the vector of available bandwidths, $P_{t}$ is the available power, and $Q_{t}$ is the vector of queue lengths at the HAPs. In practice, each agent *i* only has access to a local observation $o_{t}^{i}$ of the global state. This observation contains its local CSI $h_{t}^{i}$, its allocated bandwidth $b_{t}^{i}$, its transmit power $p_{t}^{i}$, its buffer state $q_{t}^{i}$, and the predicted CSI ${\hat{H}}_{t}^{\mathrm{pred}}$ from the channel prediction module [23]. Using predicted CSI in $o_{t}^{i}$ is important in NTNs, because it lets agents base decisions on both current and expected future channel conditions instead of only reacting to the latest measurement. In (10), $r_{i}$ denotes the reward received by agent *i*; in our design we use a shared global reward so that all agents receive the same scalar signal $r_{i}\left(t\right)=R_{t}$ defined below.

The action of agent *i* at time *t* is a vector

(12) $$a_{t}^{i}=\left[\alpha_{u,h}^{i},\beta_{h}^{i},p_{h}^{i}\right],$$

where $\alpha_{u,h}^{i}\in \left\{0,1\right\}$ indicates UE–HAP association, $\beta_{h}^{i}$ is the bandwidth fraction assigned to HAP *h*, and $p_{h}^{i}$ is the transmit power used by HAP *h* towards the LEO. The joint action $a_{t}=\left(a_{t}^{1},\ldots ,a_{t}^{N}\right)$ affects both local link performance and the global behavior of the network.

To steer learning towards cooperative and fair use of resources, we design the reward as a weighted sum of four terms:

(13) $$R_{t}=\lambda_{1}\frac{C_{t}}{C_{\max}}-\lambda_{2}\frac{D_{t}}{D_{\max}}+\lambda_{3}J_{t}+\lambda_{4}\frac{E_{t}^{\mathrm{collab}}}{E_{\max}},$$

where $C_{t}$ is the total capacity, $D_{t}$ is the average end-to-end delay, $J_{t}$ is Jain’s fairness index, and $E_{t}^{\mathrm{collab}}$ is the number of successful HAP–LEO collaboration events at time *t*. The coefficients $\lambda_{1},\lambda_{2},\lambda_{3},\lambda_{4}$ control the relative weight of each term. In our implementation, when these metrics violate preset thresholds, we convert them into penalty terms. This reduces free-riding behavior that can occur when agents only receive a shared global reward [24]. Here $C_{\max}$, $D_{\max}$, and $E_{\max}$ are normalization constants that scale the corresponding terms to comparable numerical ranges in the canonical scenario. In our experiments, we choose $\lambda_{1}=1.0$, $\lambda_{2}=0.5$, $\lambda_{3}=0.5$, and $\lambda_{4}=0.8$. We first fixed $\lambda_{1}$ as the reference scale for the capacity term and then varied $\lambda_{2}$, $\lambda_{3}$, and $\lambda_{4}$ over a small set of candidate values in preliminary runs. Very large delay and fairness weights (for example, values on the order of 1.0) made the policies overly conservative and reduced the total reward, whereas very small weights led to unstable delay and degraded fairness. The reported choice provides a balance in which none of the components dominates the reward and all four metrics improve jointly; we did not attempt an exhaustive optimization over all weight combinations. In summary, the per-slot association, power, and bandwidth limits in (2)–(4) are enforced as hard constraints through the action parameterization, while the fairness and delay requirements from (8)–(9) are treated as soft constraints via the shaped reward in (13).

**Figure 2:**
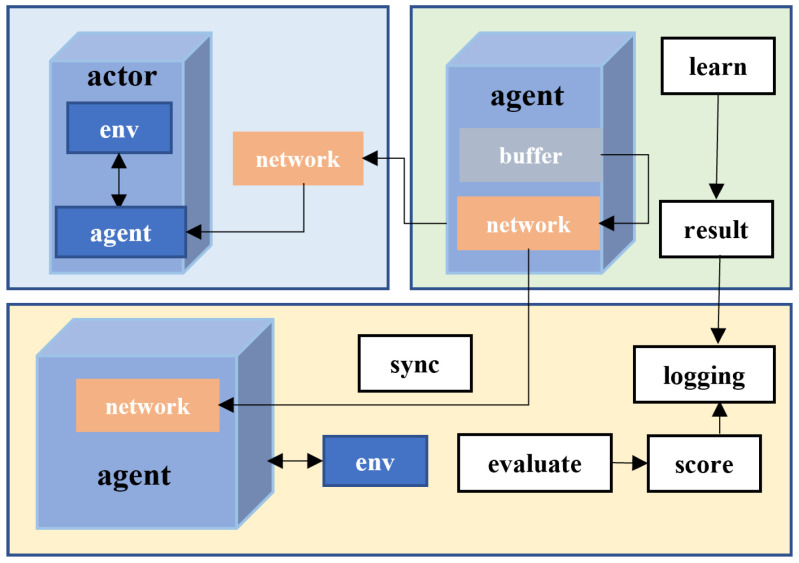
MADDPG-based multi-agent DRL framework with centralized training and decentralized execution.

We adopt the Multi-Agent Deep Deterministic Policy Gradient (MADDPG) algorithm under the CTDE paradigm. The overall MADDPG-based multi-agent DRL framework under CTDE is illustrated in Figure 2. Each agent *i* maintains a deterministic policy

(14) $$\mu_{\theta_{i}}:{𝒪}_{i}\to A_{i},$$

with parameters $\theta_{i}$, and a centralized critic

(15) $$Q_{\phi_{i}}\left(s,a\right),$$

with parameters $\phi_{i}$, that takes as input the global state s and the joint action a. The critic is trained by minimizing the temporal-difference (TD) error:

(16) $$L\left(\phi_{i}\right)=E\left[{\left(y_{i}-Q_{\phi_{i}}\left(s_{t},a_{t}\right)\right)}^{2}\right],$$

where

(17) $$y_{i}=r_{i}+\gamma Q_{\phi_{i}^{\prime }}\left(s_{t+1},a_{t+1}^{\prime }\right),\;a_{t+1}^{\prime j}=\mu_{\theta_{j}^{\prime }}\left(o_{t+1}^{j}\right),$$

and $\theta_{j}^{\prime },\phi_{i}^{\prime }$ are the parameters of the target actor and target critic networks.

The actor parameters $\theta_{i}$ are updated using the deterministic policy gradient:

(18) $$\nabla_{\theta_{i}}J\approx E\left[\nabla_{\theta_{i}}\mu_{\theta_{i}}\left(o^{i}\right)\nabla_{a^{i}}Q_{\phi_{i}}\left(s,a\right){|}_{a^{i}=\mu_{\theta_{i}}\left(o^{i}\right)}\right].$$

Training is carried out in the simulated SAGIN environment. At each time step, each agent chooses an action from its policy, receives a reward and the next observation, and stores the transition $\left(o_{t},a_{t},r_{t},o_{t+1}\right)$ in a replay buffer. We then sample mini-batches from this buffer to update the actor and critic networks by gradient descent [25]. In CTDE, the critic has access to the global state and joint action during training, which improves value estimation, while at test time each agent uses only its local observation, which keeps execution decentralized and scalable.

A key feature of our framework is the integration of the FMA channel prediction module into the DRL loop. Instead of using only instantaneous CSI, each agent augments its local observation with predicted CSI generated by the FMA model described in Section 3.4. This allows the policies to exploit temporal correlations in the channels and to adjust allocations in advance when a link is expected to improve or degrade. In our experiments, this integration reduces policy oscillations, speeds up convergence, and improves robustness under the rapid channel changes that are typical of LEO-based NTNs, leading to higher throughput and system efficiency than the tested baseline methods.

### 3.4. Fusion of Mamba and Attention Model for Channel Prediction

As discussed in Section 1, fast-varying and partially observed channels make it desirable to use predicted CSI in NTN scheduling. To this end, we build an FMA model as a sequence predictor. It is a hybrid architecture that combines a state-space backbone with an attention block. The FMA module serves as a prediction front-end for the multi-agent DRL scheduler and provides each agent with an estimate of near-future CSI. This extra information helps to reduce policy oscillations and performance drops when channels vary fast. We choose Mamba because it can model long CSI sequences with linear complexity in the sequence length and matches well the continuous-time dynamics of NTN channels.

**Figure 3:**
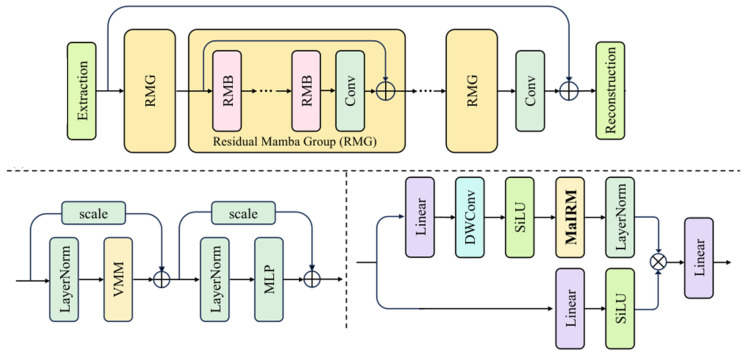
FMA model architecture combining state-space modeling and self-attention for NTN channel prediction.

As shown in Figure 3, the FMA architecture has three main parts. The first part is a Mamba state-space backbone that extracts long-term temporal features from CSI histories with linear-time complexity. The second part is a multi-head self-attention (MHSA) block that focuses on short-term variations and local structures. The third part is a fusion layer with residual connections, normalization, and a feed-forward network, which combines the two feature sets into a single predictive embedding. A final output head maps this embedding to the predicted CSI.

Formally, let $h_{t}\in R^{d}$ be the CSI vector at time slot *t*. It can include large-scale path loss, small-scale fading, Doppler shift, and shadowing. Given a history window

$$X_{t}=\left[h_{t-L+1},\ldots ,h_{t}\right],$$

the FMA model learns a mapping

$${\hat{h}}_{t+\Delta }=F_{\Theta }\left(X_{t}\right),$$

where Δ is the prediction horizon. In our implementation, we use a history length of L=10 time slots and predict Δ=3 slots ahead, which we found to offer a good trade-off between prediction accuracy and computational cost.

The Mamba part is a state-space model (SSM) with time-varying and input-dependent transition matrices:

(19) $$s_{\tau +1}=A_{\tau }s_{\tau }+B_{\tau }x_{\tau },\;z_{\tau }=C_{\tau }s_{\tau }+D_{\tau }x_{\tau }.$$

Compared with standard RNNs or CNNs, Mamba updates its state with a selective memory mechanism and has a dynamic receptive field. This produces a sequence of hidden states

$$Z_{t}=\left[z_{t-L+1},\ldots ,z_{t}\right],$$

which encodes long-range channel behavior such as orbital periodicity, slow fading trends, and repeated blockage events.

The MHSA block is then applied to $Z_{t}$. While Mamba captures global trends, it may smooth out some abrupt changes. The MHSA module computes attention weights across all time positions:

(20) $$\mathrm{Att}\left(Q,K,V\right)=\mathrm{softmax}\;\left(\frac{QK^{⊤}}{\sqrt{d_{k}}}\right)V,$$

where queries, keys, and values are linear projections of $Z_{t}$. Using multiple heads allows the model to focus on different temporal regions at the same time, for example recent Doppler spikes or fading dips. The output of this block is a feature sequence $U_{t}$ that preserves fine-grained CSI changes.

We then fuse the outputs of Mamba and MHSA:

(21) $$F_{t}=\mathrm{LayerNorm}\left(Z_{t}+U_{t}\right),$$

followed by a position-wise feed-forward network:

(22) $$F_{t}^{\prime }=\mathrm{GELU}\left(F_{t}W_{1}+b_{1}\right)W_{2}+b_{2}.$$

An attention-based temporal pooling layer then aggregates the sequence into a compact vector $f_{t}$, and the output layer predicts the future CSI as

(23) $${\hat{h}}_{t+\Delta }=W_{\mathrm{pred}}^{O}f_{t}^{⊤}+b_{\mathrm{pred}}^{O}.$$

The FMA predictor is trained with a mean squared error loss:

(24) $$L_{\mathrm{FMA}}=\frac{1}{B}\sum_{b=1}^{B}∥{\hat{h}}_{t+\Delta }^{\left(b\right)}-h_{t+\Delta }^{\left(b\right)}{∥}_{2}^{2},$$

where *B* is the batch size. In some experiments, we further fine-tune FMA together with the DRL scheduler using a combined loss

(25) $$L_{\mathrm{total}}=L_{\mathrm{DRL}}+\eta \;L_{\mathrm{FMA}},$$

where η is a weighting factor.

Compared with a Transformer predictor, whose self-attention cost is $O(L^{2})$ in the sequence length *L*, FMA keeps the Mamba backbone at O(Lh) and adds $O(HLd_{k})$ for the MHSA block. More specifically, for batch size *B* the overall time complexity of FMA is $𝒪(BLh+BHLd_{k})$ with memory cost 𝒪(BLh), since no L×L attention matrices are formed. In contrast, a vanilla Transformer-based predictor has time complexity $𝒪(BL^{2}d_{k})$ and memory complexity $𝒪(BL^{2})$ due to the quadratic self-attention. This linear dependence on *L* makes it practical to use longer CSI histories on resource-constrained HAP or LEO platforms. With the configuration in Table 1 (hidden size h=128, four state-space blocks, four attention heads, and a short history window), the resulting model has a few hundred thousand parameters and a per-step computational cost that is comparable to lightweight sequence models commonly used on embedded devices, making real-time inference on HAP or LEO payload processors feasible in principle. Long histories are useful in NTN because they contain both slow orbital trends and fast fading effects. In our design, the Mamba part mainly stabilizes predictions by modeling global patterns, while the MHSA part makes the predictor more sensitive to sudden changes. Compared with generic hybrid state-space–attention architectures, our FMA design is tailored to NTN channel prediction in three ways. First, multi-head self-attention is applied only on top of the Mamba-generated hidden sequence, rather than on the raw CSI input, so that the overall time and memory complexity remain linear in the history length. Second, an attention-based temporal pooling layer is introduced to emphasize the most relevant time steps when forecasting $h_{t+\Delta }$ under multi-scale NTN dynamics. Third, the predictor is not used in isolation but is tightly coupled with a constraint-aware MADDPG scheduler under the CTDE paradigm, so that its capacity and receptive field are chosen with the joint prediction-and-control task in mind.

When FMA is used in front of the MARL scheduler, each agent receives both current CSI and its predicted continuation in its observation vector. For example, a HAP can reduce bandwidth assigned to a link that is predicted to degrade, or postpone a high-rate transmission until a predicted Doppler peak has passed. In the experiments, such prediction-aware decisions reduce the number of times that constraints are violated in the reward function and lead to higher total reward, better system efficiency, and more frequent collaboration events compared with using instantaneous CSI alone [26].

### 3.5. FMA-MADDPG for Cooperative Scheduling

To make full use of the predicted CSI from the FMA model and to cope with the dynamic and heterogeneous nature of NTNs, we build an integrated FMA-MADDPG framework for cooperative uplink scheduling [27]. Unlike MARL schemes that rely only on instantaneous CSI, our approach feeds predicted CSI into the learning loop. In this way, agents can make scheduling decisions that take expected channel changes into account instead of reacting only to the latest measurements.

At each time slot *t*, the network collects the current system state, including CSI on the UE–HAP and HAP–LEO links $H_{t}^{\mathrm{UE}-\mathrm{HAP}}$ and $H_{t}^{\mathrm{HAP}-\mathrm{LEO}}$, the available bandwidth $B_{t}$, the available transmission power $P_{t}$, and the queue lengths $Q_{t}$ at the HAPs. For agent *i*, the initial local observation at time *t* is

(26) $$o_{t}^{i}=\left[h_{t}^{i},b_{t}^{i},p_{t}^{i},q_{t}^{i}\right],$$

where $h_{t}^{i}$ is the instantaneous CSI relevant to agent *i*, $b_{t}^{i}$ is its available bandwidth, $p_{t}^{i}$ is its available power, and $q_{t}^{i}$ is its local queue length.

At the same time, the CSI history

$$X_{t}=\left[h_{t-L+1},\ldots ,h_{t}\right]$$

is sent to the FMA predictor, which outputs predicted CSI for the next Δ time steps:

(27) $${\hat{h}}_{t+\Delta }=F_{\Theta }\left(X_{t}\right).$$

We then build an enhanced observation by concatenating current and predicted information:

(28) $${\tilde{o}}_{t}^{i}=\left[o_{t}^{i},{\hat{h}}_{t+\Delta }\right].$$

This ensures that each agent sees both the present channel state and an estimate of its near-future evolution.

Each agent feeds the enhanced observation ${\tilde{o}}_{t}^{i}$ into its actor network $\mu_{\theta_{i}}$ to obtain a composite action:

(29) $$a_{t}^{i}=\mu_{\theta_{i}}\left({\tilde{o}}_{t}^{i}\right)=\left[\alpha_{u,h}^{i},\beta_{h}^{i},p_{h}^{i}\right],$$

where $\alpha_{u,h}^{i}$ is the UE–HAP association decision, $\beta_{h}^{i}$ is the bandwidth allocation ratio, and $p_{h}^{i}$ is the power level for HAP–LEO transmission [28]. The joint action of all agents, $a_{t}=\left(a_{t}^{1},\ldots ,a_{t}^{N}\right)$, controls link usage and therefore affects system throughput, delay, and fairness. Under the CTDE setting, the critic network receives the global state $s_{t}$ and the joint action $a_{t}$ and evaluates their quality during training.

Once the actions are chosen, they are executed in the three-layer network: each UE is bound to a HAP according to $\alpha_{u,h}^{i}$, bandwidth fractions are assigned to UE–HAP links according to $\beta_{h}^{i}$, and power levels on HAP–LEO links follow $p_{h}^{i}$. This changes the system state, queue lengths, and load for the next time slot. After execution, we compute the system-level feedback and give each agent a shared reward:

(30) $$R_{t}=\lambda_{1}\frac{R_{t}^{\mathrm{tot}}}{R^{\max}}-\lambda_{2}\frac{E_{t}}{E^{\max}}-\lambda_{3}\frac{D_{t}}{D^{\max}}+\lambda_{4}\frac{C_{t}^{\mathrm{collab}}}{C^{\max}},$$

where $R_{t}^{\mathrm{tot}}$ is the total throughput at time *t*, $E_{t}$ is the energy consumption, $D_{t}$ is the average transmission delay, and $C_{t}^{\mathrm{collab}}$ is the number of successful cooperation events. The weights $\lambda_{1},\ldots ,\lambda_{4}$ balance throughput, energy–delay trade-off, load balancing, and cooperation. The terms $R^{\max}$, $E^{\max}$, $D^{\max}$, and $C^{\max}$ are corresponding normalization constants chosen according to typical values observed in the canonical scenario so that the different components of the reward have comparable scales.

The FMA-MADDPG framework can be viewed as a data flow that includes CSI measurement, prediction, observation construction, action selection, and reward feedback. Predicted CSI extends the agents’ temporal horizon, the reward design promotes cooperation, and CTDE keeps training stable even when channel conditions and topology change quickly. The reward in (30) follows the same constraint-shaped design as (13), combining throughput, energy, delay, fairness, and cooperation into a single scalar signal. Overall, we first formulate the uplink resource allocation as a mixed-integer nonlinear program with explicit association, power, fairness, and delay constraints, and then approximate it by a prediction-augmented multi-agent RL scheme where hard per-slot limits are enforced at the action level and long-term fairness and delay are encoded through a shaped reward.

## 4. Experiments and Results

This section evaluates the proposed FMA-MADDPG framework in a simulated three-tier NTN scenario. We study both the FMA channel prediction module and the cooperative scheduling performance of the multi-agent RL scheme. Each simulation run includes multiple episodes with different traffic levels and mobility patterns. We focus on several key metrics [29,30,31]: total reward, system efficiency, number of collaboration events, and load balance. Together, these metrics reflect throughput, fairness, and cooperation across the UE–HAP–LEO layers.

### 4.1. Experimental Settings

**Figure 4:**
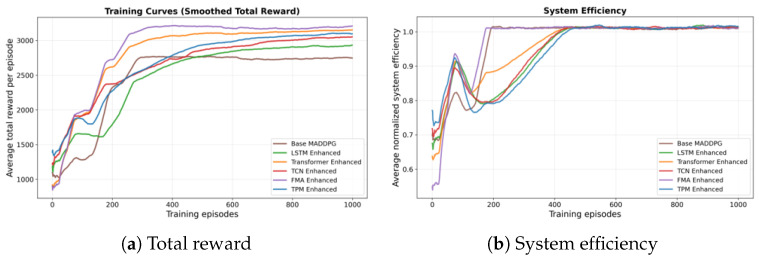
Comparison of channel prediction models under the canonical UE–HAP–LEO NTN configuration. (**a**) Average total reward per episode versus training episodes. (**b**) Average normalized system efficiency versus training episodes.

**Figure 5:**
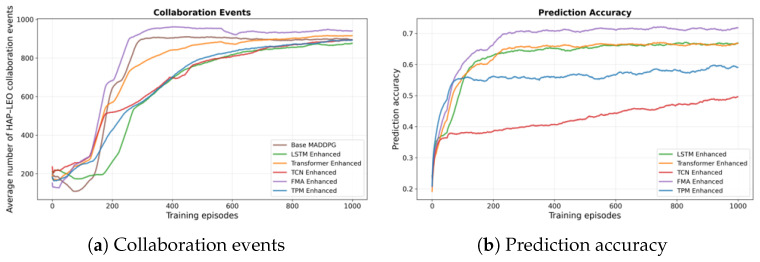
Comparison of channel prediction models under the canonical UE–HAP–LEO NTN configuration. (**a**) Average number of HAP–LEO collaboration events per episode versus training episodes. (**b**) Prediction accuracy versus training episodes.

**Figure 6:**
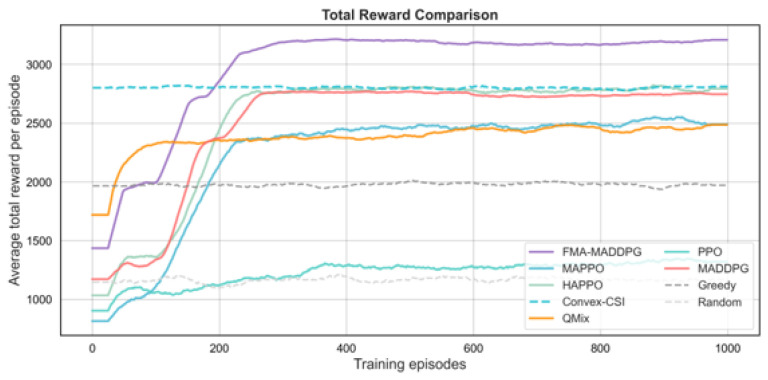
Average total reward per episode versus training episodes for different scheduling methods under the canonical UE–HAP–LEO NTN configuration.

**Figure 7:**
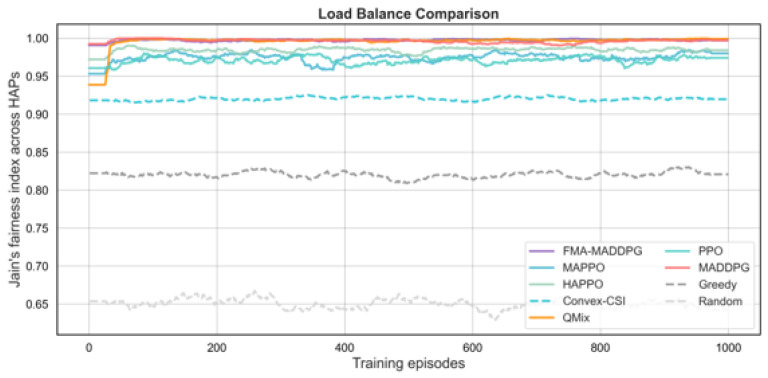
Jain’s fairness index across HAPs versus training episodes for different scheduling methods under the canonical UE–HAP–LEO NTN configuration.

To evaluate the proposed framework, we build a simulation dataset that emulates channel dynamics and traffic patterns in a three-layer SAGIN [32,33]. The dataset is generated using a Python 3.8 simulator [34]. In this environment, low-Earth-orbit (LEO) satellites follow predefined orbits and interact with HAPs and distributed UEs. The channel traces for UE–HAP links include large-scale path loss, log-normal shadowing, and Rayleigh fading. The HAP–LEO channels additionally include Doppler shifts and slowly varying shadowing caused by satellite motion. Each simulation episode produces a time series of CSI and traffic arrivals, which we use to train and test both the prediction and the scheduling modules. This setup allows controlled but still realistic tests under different traffic and mobility conditions. All results in Figure 4, Figure 5, Figure 6 and Figure 7 and Table 2 are obtained under one canonical NTN uplink configuration defined in Section 3.1 and this subsection. For each method, we train in this canonical scenario and then evaluate the learned policies over multiple test episodes with independently generated traffic and channel realizations. Due to computational constraints, we train one policy realization (i.e., one random initialization and one full training run) per learning-based method. The reported curves and scalar values are therefore averages over multiple evaluation episodes for a single trained policy per method, and we do not provide confidence intervals or statistical hypothesis tests; the results should be viewed as representative for this configuration rather than as statistically guaranteed performance bounds.

In the canonical UE–HAP–LEO scenario, each high-altitude platform (HAP) is deployed at an altitude of 20,000 m and each low-Earth-orbit (LEO) satellite orbits at 600,000 m. Every HAP has a computing capability of 8.0 GHz and can handle up to 20 concurrent tasks, while each LEO satellite has a computing capability of 15.0 GHz and a maximum load of 50 tasks. To reflect the different propagation characteristics of the links, the large-scale channel gains are initialized within predefined ranges in dB: 5–25 dB for UE–HAP links (short-range, relatively high gain), 10–30 dB for HAP–LEO links (more stable, high gain), and −5–15 dB for UE–LEO links when such links are considered [35].

For the learning setup, we use $N_{\mathrm{agents}}=4$ cooperating agents. The local observation (state) dimension is 15 and the action dimension is 4, consistent with the UE–HAP–LEO resource control problem. The actor networks follow a fully connected architecture of 15→256→128→64→4, and the critic networks use a 76→512→256→128→64→1 architecture. These sizes are chosen to provide sufficient representational power for the joint prediction-and-scheduling task while keeping the computational cost moderate for potential deployment on HAP or satellite platforms. At deployment time, only the actor networks are used for inference, so the per-step cost of the MARL policy reduces to a few fully connected layers per agent. All reported metrics in Section 4 are obtained by averaging over multiple training and evaluation episodes with independently generated channel and traffic realizations for each trained policy.

In the canonical configuration, we simulate 50 ground UEs and 3 HAPs that forward traffic to a small constellation of 4 LEO satellites. The three HAPs are deployed at the same altitude with identical hardware capabilities and roughly symmetric coverage regions. The carrier frequency used in the path-loss calculations is 2.4 GHz. The total bandwidth available for the UE–HAP links is 100 MHz, which is partitioned into multiple OFDM sub-channels. Each LEO satellite has a per-satellite bandwidth of 50 MHz for HAP–LEO transmission. User transmit powers are limited to 16 dBm, while each HAP can transmit up to 30 dBm towards the LEO layer. Traffic arrivals at UEs follow independent stochastic processes with region-dependent mean arrival rates between 0.3 and 0.6 tasks per time slot. The regional mean loads are chosen to be similar, so that the canonical scenario is approximately symmetric in both HAP capabilities and long-term traffic intensity.

For all MADDPG-based schedulers and their baselines, we use a discount factor of 0.99, an actor learning rate of $1\times 10^{-4}$, a critic learning rate of $5\times 10^{-5}$, a replay buffer with capacity 100,000 transitions, and a mini-batch size of 256. The target actor and critic networks are updated using a soft update coefficient of 0.001. Each method is trained for a fixed number of episodes and then evaluated over a separate set of episodes with independently generated channel and traffic realizations; all curves and scalar metrics reported in Section 4 are averages over these evaluation episodes.

### 4.2. Performance Comparison of Channel Prediction Models

We first study how different channel prediction models affect the NTN scheduling performance. We compare five predictors under the same MADDPG scheduler: an LSTM, a TCN, a probabilistic TPM, a transformer-based predictor, and the proposed FMA model. Figure 4a,b show total reward and system efficiency. Figure 5a,b show the number of collaboration events and the prediction accuracy.

From the results, the FMA predictor achieves a total reward of about 3200, higher than TCN (2867), LSTM (2574), TPM (2431), and the transformer-based predictor (about 3050). The total reward summarizes throughput, latency, and energy terms in the reward function, so this gain suggests that the use of predicted CSI helps agents strike a better balance between data rate and power cost.

The system efficiency for a given scheduling method *m* is defined as the average throughput per unit total bandwidth and power over an evaluation episode of length *T*:

(31) $$\eta_{\mathrm{sys}}^{\left(m\right)}=\frac{1}{T}\sum_{t=1}^{T}\frac{C_{t}^{\left(m\right)}}{B_{\mathrm{Tot}}P_{\mathrm{Tot}}},$$

where $C_{t}^{\left(m\right)}$ is the total system throughput at time slot *t* under method *m*, and $B_{\mathrm{Tot}}$ and $P_{\mathrm{Tot}}$ denote the total available bandwidth and the total maximum transmit power in the canonical UE–HAP–LEO scenario, respectively. Since $\eta_{\mathrm{sys}}^{\left(m\right)}$ is a throughput-per-resource ratio rather than a probability or a normalized cumulative distribution, it is not mathematically restricted to the interval [0,1]. Values smaller than, equal to, or larger than one simply correspond to lower, equal, or higher levels of achieved bits per second per unit of aggregated bandwidth and power in the considered setting. The transformer-based model attains an efficiency close to 1.0 in the canonical scenario, while FMA reaches 0.95 and the LSTM and TPM baselines remain below 0.85. Since this metric is a throughput-per-resource ratio rather than a quantity normalized to a fixed upper bound, it is not restricted to the interval [0,1]; values slightly below or above one correspond to lower or higher levels of bits per second achieved per unit of (Hz·W). This means that prediction-aware schedulers use the same resources more effectively when longer temporal dependencies are exploited. The improvement is consistent across episodes and indicates that temporal foresight stabilizes scheduling decisions under fast channel variations.

The number of successful collaboration events between HAPs and LEOs reaches 956 under FMA, which is higher than the transformer-based predictor (about 915) and about 13.5% higher than the best non-transformer baseline. More collaboration events show that predicted CSI gives agents better awareness of cross-layer link conditions, so they can coordinate power control and association more often. FMA also attains a prediction accuracy of 0.78, compared with about 0.67 for the Transformer-based predictor, 0.64 for LSTM, 0.71 for TCN, and 0.68 for TPM. Since the Transformer serves as an attention-only baseline, this comparison supports the idea that combining Mamba’s state-space modeling with multi-head attention helps capture both long-term orbital patterns and short-term fading changes better than attention-only or purely recurrent/convnet-based models. A more fine-grained architectural ablation that explicitly isolates Mamba-only and attention-only variants of FMA is left for future work.

All five predictors in this subsection are paired with the same MADDPG-based scheduler and evaluated under the same canonical scenario, so the absolute total reward of the FMA-based configuration here matches the FMA-MADDPG result reported in Section 4.3. Overall, these results show that accurate and stable channel prediction is an important component for intelligent scheduling. By adding predicted CSI to the observations, FMA not only improves steady-state performance, but also makes the training process more stable, leading to smoother and more reliable learning curves.

### 4.3. Comparison with Other Deep Reinforcement Learning Frameworks

**Figure 8:**
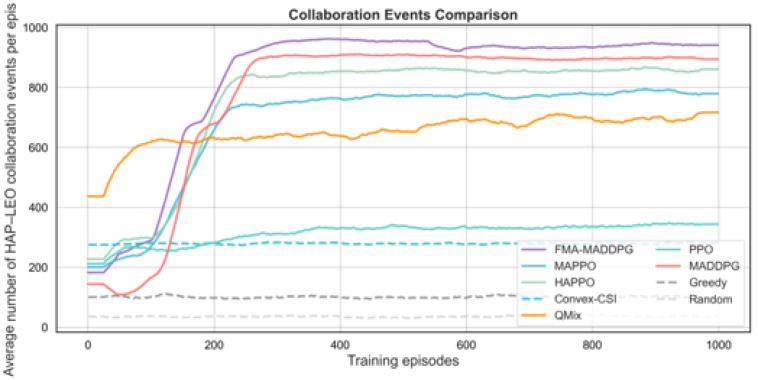
Average number of HAP–LEO collaboration events per episode versus training episodes for different scheduling methods under the canonical UE–HAP–LEO NTN configuration.

**Figure 9:**
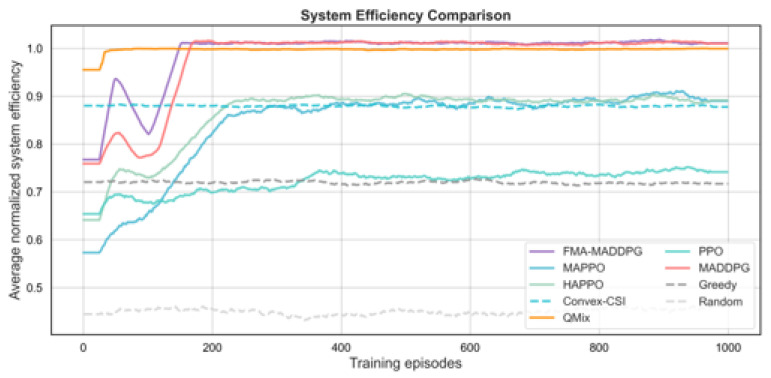
Average normalized system efficiency versus training episodes for different scheduling methods under the canonical UE–HAP–LEO NTN configuration.

We next compare FMA-MADDPG with several multi-agent RL baselines (PPO, MAPPO, HAPPO, QMIX, and standard MADDPG) and with two non-RL baselines and a random scheduler. The first non-RL baseline, denoted Convex-CSI, approximates the per-slot joint association and power allocation under perfect instantaneous CSI by solving a convex-relaxed version of the uplink resource allocation problem. The second, denoted Greedy, is a myopic heuristic that associates each UE to the HAP with the best instantaneous channel quality, allocates bandwidth in proportion to instantaneous rates, and distributes transmit power according to a simple rule, without learning. We also include a purely random scheduler that selects feasible associations and power levels uniformly at random. All methods use the same network configuration and traffic settings for a fair comparison. The results in Figure 6, Figure 7, Figure 8 and Figure 9 and Table 3 show that FMA-MADDPG achieves higher performance than these baselines on most metrics in our experiments under the canonical scenario. All schedulers compared in this subsection share the same canonical NTN configuration as in Section 4.2; therefore, the FMA-MADDPG score refers to the same setting and should be interpreted in terms of relative differences to the other schedulers.

In terms of total reward, FMA-MADDPG achieves the highest average value of 3199.4, which is higher than HAPPO (2789.3), MADDPG (2749.1), MAPPO (2519.3), QMIX (2465.4), and PPO (1331.1). Among the non-RL baselines, Convex-CSI reaches an average reward of 2812.7, which is clearly better than the greedy heuristic (1974.6) and the random scheduler (1160.8) but still below the best RL-based methods. This shows that combining predicted CSI with a constraint-based cooperative scheduler can improve the overall return beyond both heuristic and convex-relaxation approaches. The number of collaboration events between the HAP and LEO layers is also highest for FMA-MADDPG (942 on average), followed by MADDPG (897) and HAPPO (858), while Convex-CSI (282), Greedy (100), and Random (36) exhibit much less frequent cooperation. This indicates that the prediction-aware MARL schemes are better able to exploit cross-layer links for coordination than the non-learning baselines in the dynamic NTN setting.

For load balance, FMA-MADDPG and MADDPG both achieve Jain’s indices close to one (0.997), while Convex-CSI and Greedy produce lower fairness values (0.920 and 0.825, respectively). These near-unity indices indicate very even traffic distribution among HAPs in the canonical scenario and are partly a consequence of the deliberately symmetric configuration, where HAPs have identical capabilities and regions have similar average traffic. Under such symmetric conditions and with a fairness-aware reward, Jain’s indices close to 1.0 are expected and should not be interpreted as typical for arbitrary NTN deployments. In more heterogeneous or larger-scale settings with asymmetric HAP capacities, more uneven user distributions, or different link budgets, we would expect lower fairness indices in absolute value, although we still anticipate that FMA-MADDPG would retain advantages relative to the baselines. In terms of system efficiency, FMA-MADDPG and MADDPG both attain values slightly above one (1.011 and 1.012, respectively), while Convex-CSI and HAPPO reach around 0.88–0.89 and the Greedy and Random baselines achieve much lower efficiencies (0.718 and 0.457). As discussed in (31), this metric is a linear throughput-per-resource measure and is therefore not mathematically bounded by one; values greater than one simply indicate that these schedulers achieve more than one unit of normalized throughput per unit of aggregated bandwidth and power in the canonical scenario. These results confirm that FMA-MADDPG delivers high throughput and efficient use of resources while maintaining fairness and cooperation advantages over both RL and non-RL alternatives in this canonical scenario.

### 4.4. Ablation Study on Prediction and Constraint Design

To further isolate the contributions of the channel prediction module and the constraint-aware reward, we conduct an ablation study under the same canonical UE–HAP–LEO configuration. We compare four variants built on the same MADDPG backbone: (A) a constrained scheduler without prediction, which uses the full structured reward but only instantaneous CSI; (B) an unconstrained scheduler without prediction, which removes the constraint penalties and fairness/cooperation terms; (C) an unconstrained scheduler with prediction, which removes the constraint penalties but augments the agents’ observations with predicted CSI; and (D) the full FMA-MADDPG scheme with both prediction and constraints.

**Figure 10:**
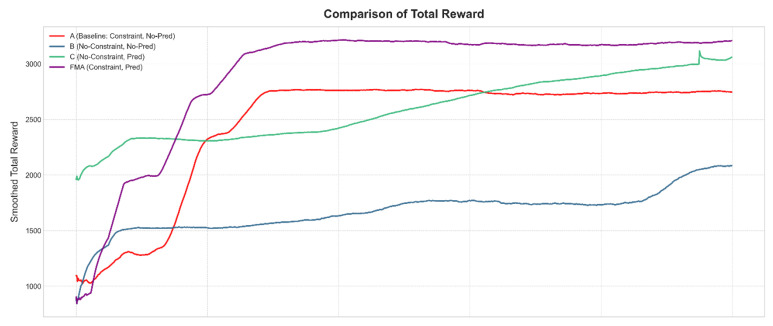
Ablation study of prediction and constraint design under the canonical UE–HAP–LEO NTN configuration: average total reward per episode versus training episodes for different variants.

**Figure 11:**
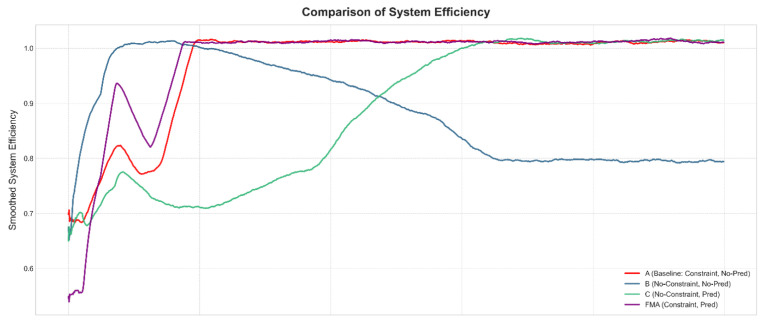
Ablation study of prediction and constraint design under the canonical UE–HAP–LEO NTN configuration: average normalized system efficiency per episode versus training episodes for different variants.

**Figure 12:**
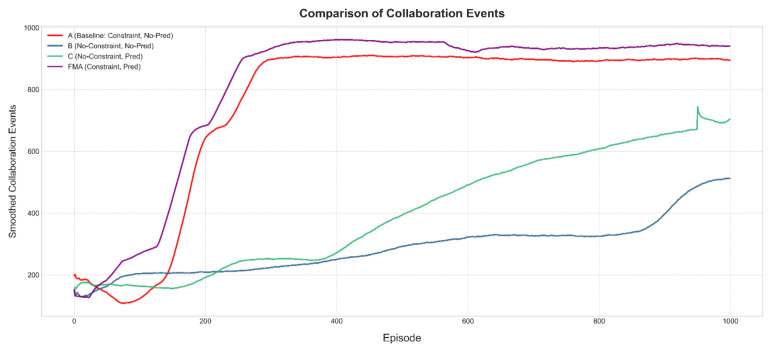
Ablation study of prediction and constraint design under the canonical UE–HAP–LEO NTN configuration: average number of HAP–LEO collaboration events per episode versus training episodes for different variants.

Figure 10, Figure 11 and Figure 12 illustrate the learning curves of these four variants in terms of total reward, system efficiency, and collaboration events. The unconstrained and non-predictive variant (B) yields the lowest average total reward, around 2100, together with a degraded system efficiency of about 0.78 and roughly 450 collaboration events per episode. This behavior indicates that removing the constraint terms leads to unstable policies that can exploit the reward in the short term but eventually drift towards inefficient and poorly coordinated operation. Adding only the constraint-aware reward while still relying on instantaneous CSI (variant A) increases the average total reward to about 2400, maintains system efficiency around 1.0, and nearly doubles the number of collaboration events to about 900, showing that the constraint mechanism plays a key role in stabilizing learning and promoting cooperation even without prediction.

Introducing only the prediction module (variant C) produces a much larger improvement. In this case, the total reward rises to about 3000, the system efficiency recovers to approximately 1.0 from the degraded level seen in the unconstrained baseline, and the number of collaboration events increases to around 700. This demonstrates that predicted CSI by itself brings a substantial gain in both performance and coordination, as it allows agents to anticipate channel variations and to allocate resources more proactively. Finally, combining both prediction and constraints in the full FMA-MADDPG configuration (variant D) yields the best results: the total reward reaches about 3200, the convergence is the fastest among all variants, the system efficiency stabilizes slightly above 1.0, and the number of collaboration events approaches 950. Overall, these ablation results indicate that the constraint-aware reward is essential for stability and fairness, the prediction module provides a large boost in reward and efficiency, and their combination in FMA-MADDPG delivers the most stable and effective behavior. Together with the comparison to the Transformer-based predictor in Section 4.2, they suggest that both the presence of prediction and the specific Mamba + attention design matter for performance. A more detailed architectural ablation that separates Mamba-only and attention-only versions of FMA will be considered in future work.

## 5. Discussion

This section interprets the experimental results and explains how the FMA-MADDPG framework helps address key challenges in NTNs. This section interprets the experimental results and explains how the main components of FMA-MADDPG contribute to the observed improvements in total reward, collaboration frequency, load balance, and system efficiency. We discuss the roles of the prediction module, the reinforcement learning design, and their joint effect at the system level.

### 5.1. Impact of Channel Prediction on System Performance

Accurate channel prediction is important for link stability and resource allocation in NTNs, where channels change with user positions, weather, and satellite motion. In our experiments, the FMA predictor attains a prediction accuracy of 0.78, higher than the LSTM, TCN, and TPM baselines. This gain in prediction accuracy is reflected in the higher total reward and system efficiency, because agents with predicted CSI can anticipate link degradation and adjust power and bandwidth before performance drops.

The FMA module uses a Mamba state-space backbone together with multi-head self-attention. The state-space part captures long-term patterns, such as orbital periodicity and slow fading trends. The attention part focuses on short-term changes, such as Doppler fluctuations and sudden interference. In combination, these two parts produce predictions that are neither too smooth nor too noisy. This helps to reduce oscillations in the learned policies and improves convergence during training.

From a network operation point of view, better prediction allows UEs and HAPs to schedule more proactively. For example, a HAP can reduce bandwidth to a link that is predicted to experience deep fading in the next slots, or it can delay non-urgent transmissions when a Doppler peak is expected. Such decisions lead to smoother use of links, fewer retransmissions, and more balanced service, which is consistent with the observed load-balance index of 1.00 across HAPs. In this sense, channel prediction acts both as a performance booster and as a stabilizing factor for multi-agent coordination under time-varying conditions. For remote-sensing and Earth observation applications, higher throughput and lower delay directly improve the timeliness of sensing data delivery. They also enhance the reliability of coverage for missions such as disaster monitoring and environmental surveillance. These tasks represent typical service scenarios targeted by large-scale NTN deployments.

### 5.2. Effectiveness of Reinforcement Learning and Reward Design

The reinforcement learning part of FMA-MADDPG is designed to deal with two issues that often appear in MARL for NTNs: unstable convergence and free-riding. To this end, we use a reward that combines throughput, energy, delay, and cooperation, and that turns some metrics into penalties when they violate thresholds. This guides agents towards global objectives while still keeping local fairness in mind.

Concretely, the reward includes terms for capacity, average end-to-end delay, Jain’s fairness index, and the number of collaboration events. When delay is too high or fairness drops below a target, penalty terms are added. This reduces the misalignment that can appear in simple shared-reward schemes, where some agents might benefit from others’ actions without contributing much themselves.

The experimental results summarized in Table 3 support this design. FMA-MADDPG achieves the highest total reward and the largest number of collaboration events among all compared methods, indicating a high level of inter-agent coordination, especially between the HAP and LEO layers. At the same time, it maintains near-perfect load balance and high system efficiency, which means that resources are well used and traffic is spread evenly across HAPs. Taken together, these observations suggest that the constraint-aware reward formulation improves both stability and fairness under decentralized execution.

In preliminary sensitivity tests, we also varied the weights $\lambda_{2}$–$\lambda_{4}$ while keeping $\lambda_{1}$ fixed, to gain intuition about the effect of the constraint terms. Reducing the fairness weight $\lambda_{3}$ shifted the learned policies towards slightly higher total reward but visibly lower Jain’s fairness indices, indicating a more throughput-oriented behavior. Increasing $\lambda_{3}$ had the opposite effect, improving fairness at the cost of a small reduction in total reward and fewer collaboration events. Similarly, strengthening the delay penalty $\lambda_{2}$ led to lower average end-to-end delay but a modest loss in throughput, while too small a value of $\lambda_{2}$ made the delay distributions broader. These qualitative trends are consistent with the intended role of the weights as knobs that trade off throughput against delay, fairness, and cooperation; a full quantitative study of this trade-off is left for future work.

The CTDE setting also plays an important role. During training, the critics can use the full global state and joint action, which leads to better value estimates. At test time, each actor only needs its own local observation, which keeps the solution scalable and practical in large NTN systems where full central control is not realistic.

### 5.3. Overall Comparison and System-Level Insights

When compared with other MARL methods such as PPO, MAPPO, HAPPO, QMIX, and standard MADDPG, as well as with the Convex-CSI and Greedy non-learning baselines, the FMA-MADDPG framework performs better on most of the evaluated metrics, as summarized in Table 3. PPO and MAPPO achieve only moderate rewards, HAPPO and standard MADDPG come closer but still fall short in terms of total reward and collaboration frequency, and QMIX attains good load balance but a lower total reward. The non-RL baselines, in particular the greedy heuristic and the random scheduler, exhibit significantly lower reward, efficiency, and cooperation levels, which underlines the advantage of prediction-augmented multi-agent RL in this dynamic NTN setting.

In contrast, FMA-MADDPG consistently improves total reward, load balance, and collaboration events. The prediction module helps bridge the gap between purely reactive control and proactive scheduling. The constraint-based reward ensures that the learned policies remain cooperative and fair, not just throughput-oriented. Together, these elements allow the framework to handle the complex and non-stationary conditions of the three-layer UE–HAP–LEO system.

Another observation is that FMA-MADDPG converges faster than the baselines. The availability of predicted CSI reduces uncertainty in the state transitions seen by the RL agents. As a result, the value function and policies stabilize more quickly, which shortens training time and can make deployment easier in practice.

We also note that, for each learning-based method, only a single policy realization is trained in the canonical NTN scenario due to the computational cost of joint prediction-and-scheduling experiments with multiple baselines. As a result, we do not report confidence intervals or statistical significance tests across random seeds. The curves and scalar metrics in Section 4 should be interpreted as representative examples under the given configuration, rather than as statistically guaranteed performance bounds. Extending the evaluation to multiple seeds with uncertainty quantification is left for future work.

From a computational perspective, the FMA predictor and the MADDPG policies were configured to be lightweight. The FMA model uses a hidden size of 128 with four state-space blocks and four attention heads, resulting in a few hundred thousand parameters and linear-time complexity in the history length, while each actor is a small fully connected network with four layers. Training is performed offline on the ground, and only inference of the FMA predictor and the actor networks would run on HAP or LEO hardware. Given these model sizes and the linear-time design, real-time inference at typical scheduling rates appears feasible for current HAP/LEO payload processors in principle, especially when using standard optimizations such as mixed-precision or fixed-point implementations. However, we have not yet measured end-to-end latency on specific hardware platforms, and a detailed hardware-in-the-loop evaluation is left for future work.

Beyond computational issues, several modeling assumptions may also affect how the framework behaves when they are relaxed. The assumption of static UEs removes Doppler effects due to user motion and keeps the UE–HAP geometry fixed within an episode; with mobile users we would expect faster variations in link quality and queue lengths, more frequent handovers, and a tighter requirement on the predictor to track these changes. The symmetric HAP deployment and approximately balanced regional traffic mainly influence the absolute level of the fairness indices: in more heterogeneous deployments with different HAP altitudes or capacities we would expect lower Jain’s indices, although the relative ordering between methods is likely to persist. Finally, the assumption of accurate historical CSI is probably the most critical for prediction quality; if the CSI fed to the FMA module is noisy or delayed, prediction accuracy and, in turn, scheduling performance can degrade. Studying robustness to user mobility, imperfect and delayed CSI, and heterogeneous HAP deployments is therefore an important part of our future work.

In future work, several promising directions will be pursued to further enhance the applicability and robustness of the proposed framework. First, real-world deployment and hardware-in-the-loop experiments will be conducted to validate the system performance under practical NTN conditions, including hardware impairments, synchronization latency, and imperfect CSI feedback. Second, the current FMA predictor can be extended toward adaptive or hierarchical structures, enabling dynamic adjustment of prediction horizons based on mobility levels or environmental variations. Third, lightweight model compression, pruning, or knowledge distillation techniques will be explored to reduce computational overhead, facilitating deployment in resource-constrained HAP or satellite platforms. Finally, integration with cross-layer optimization, network slicing, and semantic communication frameworks may further improve the intelligence and flexibility of resource management in large-scale 6G NTN ecosystems.

## 6. Conclusions

This paper proposed an FMA-MADDPG framework for uplink resource scheduling in NTNs. The framework combines channel prediction with constraint-based cooperative reinforcement learning. It is designed for a three-layer space–air–ground architecture and targets practical issues in large NTN systems, such as time-varying channels, heterogeneous traffic, and partial observability.

The core component is the FMA predictor. It uses a Mamba state-space backbone to model long-term temporal dependencies and a multi-head self-attention block to capture short-term fluctuations. This hybrid design provides accurate and stable channel forecasts and gives agents advance knowledge of link variations. By embedding predicted CSI into the observation space, the framework allows agents to make scheduling decisions that are not only based on the current state but also on expected future conditions.

On top of this prediction module, we design a constraint-based MADDPG strategy. The reward function jointly considers throughput, latency, energy efficiency, and fairness. Unlike standard multi-agent RL approaches that only share a global reward, our formulation introduces explicit performance thresholds and anti-idle penalties. This reduces free-riding behavior and encourages more balanced resource use across agents in different layers.

Simulations in a customized NTN environment show that, in our experiments under the canonical scenario, the proposed FMA-MADDPG method achieves higher performance than several baseline algorithms, including PPO, MAPPO, HAPPO, QMIX, and standard MADDPG. Under this canonical scenario, the framework attains the highest total reward, system efficiency, and number of HAP–LEO collaboration events among all compared methods, while maintaining a load-balance index very close to one. These results support the effectiveness of combining prediction with constraint-aware cooperative learning to obtain stable, fair, and efficient scheduling under dynamic network conditions. We note that the current evaluation is limited to one canonical NTN uplink scenario with static UEs, symmetric HAP deployment, and simplified channel models, and assumes accurate historical CSI; it does not yet examine scalability with respect to the number of UEs, HAPs, and LEO satellites, or robustness under blocked links, noisy CSI, and different Doppler regimes. Studying more diverse network settings, mobile users, standardized NTN channels, imperfect CSI feedback, and these scalability and robustness aspects will be part of our future work.

Overall, the FMA-MADDPG framework offers a scalable approach to resource management in future 6G space–air–ground integrated networks. Its use of prediction and cooperative decision making makes it suitable for mission-critical remote sensing applications, such as disaster monitoring, environmental observation, and intelligent navigation. In future work, we plan to extend this framework to real-world NTN testbeds, include more explicit multi-objective optimization for cross-layer control, and explore lightweight implementations for edge intelligence on HAP and satellite platforms. 

## Figures and Tables

**Table 1:** Layer configuration of the FMA channel prediction model.

Module	Layer Type	Configuration	Output
Input Encoding	Linear + Positional Embedding	d→h	L×h
Mamba Backbone	State-Space Block (×4)	Hidden size h=128	L×h
MHSA	Multi-Head Self-Attention	H=4, $d_{k}=32$	L×h
Fusion	Residual + LayerNorm	Skip connection $Z_{t}+U_{t}$	L×h
Feed-Forward	Two-layer MLP	h→2h→h, GELU	L×h
Temporal Pooling	Attention Pooling	Weight vector $w_{p}$	1×h
Output Head	Linear Projection	h→d	1×d

**Table 2:** Main notations used in the system model and optimization framework.

Symbol	Description
Z, *Z*	Set and number of ground UEs
H, *H*	Set and number of HAPs
L, *L*	Set and number of LEO satellites
A, *A*	Set and number of sub-areas
*k*, *t*	Time-slot index in an episode
$d^{\mathrm{UE}}\left(k\right)$	3D position of a UE at slot *k*
$d^{\mathrm{HAP}}$	3D position of a HAP
$B_{\mathrm{Tot}}^{\mathrm{UE}–\mathrm{HAP}}$	Total bandwidth of the UE–HAP uplink
$C_{Z}$, $|C_{Z}|$	Set and number of UE–HAP sub-channels
$B_{z,h}\left(k\right)$	Bandwidth allocated to UE *z*–HAP *h* link at slot *k*
$P_{z}^{\max}$	Maximum transmit power of UE *z*
$P_{\left(z,h\right)}\left(k\right)$	Transmit power from UE *z* to HAP *h* at slot *k*
$\varphi_{h,z}\left(k\right)$	Binary UE–HAP association indicator at slot *k*
$\theta_{\left(z,h\right)}\left(k\right)$	Instantaneous SNR on UE *z*–HAP *h* link at slot *k*
$\Phi_{\left(z,h\right)}\left(k\right)$	Instantaneous rate on UE *z*–HAP *h* link at slot *k*
${\hat{\Gamma }}_{z}\left(k\right)$	Cumulative throughput of UE *z* up to slot *k*
$I_{f}\left(k\right)$	Jain’s fairness index over all UEs at slot *k*
$\varpi_{\left(z,h\right)}\left(k\right)$	End-to-end delay of UE *z*–HAP *h* transmission at slot *k*
$R_{\mathrm{tot}}\left(k\right)$	Composite system reward at slot *k*
$H_{t}^{\mathrm{UE}–\mathrm{HAP}}$	CSI matrix of UE–HAP links at time *t*
$H_{t}^{\mathrm{HAP}–\mathrm{LEO}}$	CSI matrix of HAP–LEO links at time *t*
$B_{t}$	Vector of available bandwidths at time *t*
$P_{t}$	Vector of available transmit powers at time *t*
$Q_{t}$	Vector of HAP queue lengths at time *t*
$s_{t}$	Global system state at time *t*
$o_{t}^{i}$	Local observation of agent *i* at time *t*
$a_{t}^{i}$	Action of agent *i* at time *t*
$\alpha_{u,h}^{i}$	UE–HAP association decision in agent *i*’s action
$\beta_{h}^{i}$	Bandwidth allocation ratio to HAP *h* in agent *i*’s action
$p_{h}^{i}$	Transmit power of HAP *h* in agent *i*’s action
$C_{t}$	Total capacity (throughput) at time *t*
$D_{t}$	Average end-to-end delay at time *t*
$J_{t}$	Jain’s fairness index at time *t*
$E_{t}^{\mathrm{collab}}$	Number of collaboration events at time *t*
$\lambda_{1},\lambda_{2},\lambda_{3},\lambda_{4}$	Weights of reward components in (13) and (30)
$h_{t}$	CSI feature vector at time slot *t* (FMA input)
$X_{t}$	History window of CSI features used by FMA
*L*	Length of the CSI history window
Δ	Prediction horizon (number of slots ahead)
$Z_{t}$	Hidden sequence produced by the Mamba backbone
$U_{t}$	Feature sequence output by the MHSA block
$F_{t}$, $F_{t}^{\prime }$	Fused feature sequences before and after feed-forward network
$f_{t}$	Pooled feature vector used for CSI prediction
${\hat{h}}_{t+\Delta }$	Predicted future CSI vector at time t+Δ

**Table 3:** Summary of key performance metrics under different frameworks.

Deep Reinforcement Learning/Baseline Method	Total Reward	Collaboration Events	System Efficiency	Load Balance
PPO	1331.1	344	0.745	0.975
MAPPO	2519.3	784	0.900	0.981
HAPPO	2789.3	858	0.891	0.985
QMIX	2465.4	701	0.999	0.999
MADDPG	2749.1	897	1.012	0.997
Convex-CSI	2812.7	282	0.879	0.920
Greedy	1974.6	100	0.718	0.825
Random	1160.8	36	0.457	0.647
FMA-MADDPG (proposed)	**3199.4**	**942**	**1.011**	**0.997**

## Data Availability

The raw data supporting the conclusions of this article will be made available by the authors on request.
